# Qiancao (*Rubia* spp.) – a comprehensive metabolomics analysis of the five core species

**DOI:** 10.3389/fphar.2025.1541994

**Published:** 2025-08-18

**Authors:** Mingtong Zhang, Junyan Du, Donghua Li, Xiao Ma, Wanjun Jin, Lei Xiang, Meng Wen

**Affiliations:** ^1^ Gansu Provincial Engineering Laboratory of Chinese and Tibetan Medicine Inspection and Testing, State Key Laboratory for Quality Control of Chinese Medicinal Materials and Decoction Pieces, Gansu Institute for Drug Control, Lanzhou, China; ^2^ Department of Health, Lanzhou Modern Vocational College, Lanzhou, China; ^3^ Department of Inpatient Pharmacy, Affiliated Hospital of Chifeng University, Chifeng, China; ^4^ Department of Nursing, Gansu Health Vocational College, Lanzhou, China

**Keywords:** metabolomics, qiancao, differential accumulated metabolites, different species, discrimination

## Abstract

**Introduction:**

Qiancao comprises five closely related species: *Rubia cordifolia* L., *Rubia yunnanensis* Diels., *Rubia wallichiana* Decne., *Rubia schumanniana* Pritz., and *Rubia magna* P. G. Xiao. Due to their similar morphological characteristics, alternative species are frequently substituted for *R. cordifolia* L. in traditional medicine. However, the latest National Pharmacopoeia of China only exclusively recognizes *R. cordifolia* L. as the official medicinal source. The metabolic differences among these species remain poorly characterized, largely owing to the absence of systematic metabolomic studies.

**Methods:**

This study employed a metabolomics approach to investigate the five *Rubia* species. The selected species were analyzed using an untargeted metabolomic approach based on ultrahigh-performance liquid chromatography coupled with tandem mass spectrometry (UHPLC-MS/MS). Subsequently, multivariate statistical analysis combined with bioinformatics was applied to identify differential metabolites and elucidate their associated metabolic pathways.

**Results and discussion:**

This metabolomics analysis identified a total of 1,440 metabolites. Comparative analyses revealed significant differences in the metabolic profiles among the five species. Specifically, we detected 237, 217, 448, and 226 differentially accumulated metabolites when comparing *R. cordifolia* L. with *R. magna* P. G. Xiao, *R. cordifolia* L. with *R. schumanniana* Pritz., *R. cordifolia* L. with *R. yunnanensis* Diels., and *R. cordifolia* L. with *R. wallichiana* Decne., respectively. KEGG pathway analysis demonstrated substantial involvement of these metabolites in three key pathways: amino acid metabolism, riboflavin metabolism, and terpenoid biosynthesis, with particular emphasis on specific metabolites within these pathways. These findings provide a foundation for future research focused on species identification, quality control, biosynthetic pathway investigation, and bioactivity assessment of Qiancao, thereby advancing the understanding of its clinical applications in traditional Chinese medicine.

## 1 Introduction

The *Rubiaceae* family encompasses approximately 450 genera and 6,500 species, including diverse trees, shrubs, and botanical drug plants (González-Castelazo et al., 2023; Razafimandimbison et al., 2022; Villalobos-Flores et al., 2022). The *Rubia* genus, a member of this family, contains approximately 60 species, among which *Rubia cordifolia* L. stands out as a prominent perennial botanical drug characterized by elongated, cylindrical, and reddish roots (Murthy et al., 2022; Wen et al., 2022). This species has a wide distribution, spanning Africa, India, Malaysia, China, Japan, and Australia (Wang et al., 2023). In China, the roots of *R. cordifolia* L., locally referred to as “Qiancao”, are predominantly found in the provinces of Shaanxi, Henan, Anhui, Hebei, Shandong, Jiangsu, and Zhejiang (Wang A. K. et al., 2020). In traditional Chinese medicine, Qiancao is valued for its bitter flavor and cooling attributes, which are believed to disperse pathogenic heat from the blood, resolve blood stasis, unblock meridians, and arrest bleeding (Wang K. et al., 2020). It serves as a potent hemostatic agent, effective against hematemesis, metrorrhagia, epistaxis, as well as traumatic injuries (Chandrashekar et al., 2018; Gao et al., 2023). Beyond its medicinal applications, Qiancao also functions as an important source of natural dyes for various industries (Mijas et al., 2022). Phytochemical studies have identified over 100 distinct metabolites in Qiancao, primarily including anthraquinones, naphthoquinones, their glycosides, bicyclic hexapeptides, triterpenoids, and polysaccharides (Hitotsuyanagi et al., 2019; Humbare et al., 2022; Luo et al., 2022; Zhang et al., 2024; Zheng et al., 2017). Pharmacological research has demonstrated that Qiancao exhibits a broad spectrum of bioactivities, such as anti-inflammatory, anticancer, antioxidant, antimicrobial, antiplatelet, nephroprotective, anti-urolithiatic, hepatoprotective, and neuroprotective effects (Bana et al., 2023; Cheng et al., 2024; Liu X. et al., 2023; Qin et al., 2022; Wang et al., 2017; Zeng et al., 2023).

The genus Rubia includes five closely related species used as Qiancao, namely, *Rubia yunnanensis* Diels. (Xiaohongshen) (Zhao et al., 2022), *Rubia wallichiana* Decne. (Zangqiancao) (Wu et al., 2003), *R. cordifolia* L. (Qiancao), *Rubia schumanniana* Pritz. (Dayeqiancao), and *Rubia magna* P. G. Xiao (Daqiancao) (Li et al., 2023). Current research indicates marked variations in the metabolites among these Qiancao species (Shan et al., 2016; Wen et al., 2022). These metabolites differences likely result in varying pharmaceutical efficacies among different Qiancao species, highlighting the critical need for precise identification and differentiation methods. To date, no reliable methods have been established for distinguishing these closely related species. A systematic comparative analysis of the metabolites across these five species would provide valuable insights for species selection and resource utilization. In recent years, metabolomics has emerged as a powerful tool in food and agricultural research (Liu J. et al., 2023). Untargeted metabolome analysis using liquid chromatography coupled with high-resolution mass spectrometry enables more accurate detection of precursor ions, thereby enhancing metabolites identification compared to low-resolution techniques (Bao et al., 2022). This approach has gained increasing application in investigating metabolite variations in traditional Chinese medicines, particularly those influenced by factors including cultivars, geographical origins, cultivation practices, and storage conditions (Bao et al., 2022; Niu et al., 2022). Through advanced metabolomic approaches, we can elucidate the distinctive metabolic profiles of these Qiancao species. Such analyses not only enable accurate species authentication but also reveal potential bioactive metabolites, thus facilitating evidence-based applications in both traditional and modern medical practices.

This research employed a metabolomic approach integrating ultra performance liquid chromatography-tandem mass spectrometry (UPLC-MS/MS) with multivariate statistical analyses, incorporating both univariate analysis and chemometric methods, to systematically characterize metabolites and discriminate among five Qiancao species. The main objectives were to investigate the metabolic uniqueness of the various Qiancao species and to develop a robust classification and identification model based on metabolomic data. Additionally, KEGG pathway analysis was performed to identify the metabolic differences among these species. These findings establish a scientific foundation for quality control and standardized evaluation of differentiated Qiancao species.

## 2 Materials and methods

### 2.1 Chemicals and samples

Methanol (LC-MS grade), 2-Propanol (LC-MS grade) and acetonitrile (LC-MS grade) were purchased from CNW Technologies (Dusseldorf, Germany). Acetic acid (LC-MS grade) was purchased from Sigma Chemical Co. (St. Louis, United States). Hydrochloric acid (analytical reagent) was purchased from Shanghai Titan Technology Co., LTD. (Shanghai, China). ddH2O was purchased from AS Watson & Company Limited (Guangzhou, China). Samples of *R. cordifolia* L. [*Rubiaceae*; *Rubiae* radix et rhizoma] (Qiancao), *R. yunnanensis* Diels. [*Rubiaceae*; *Rubiae yunnanensis* radix et rhizoma] (Xiaohongshen), *R. wallichiana* Decne. [*Rubiaceae*; *Rubiae* radix et rhizoma] (Zangqiancao), *R. schumanniana* Pritz. [*Rubiaceae*; *Rubiae schumannianae* rhizoma] (Dayeqiancao), and *R. magna* P. G. Xiao [*Rubiaceae*; *Rubiae magna* radix et rhizoma] (Daqiancao) were authenticated by Chief Chinese pharmacist Xiao Ma from the Gansu Institute for Drug Control. Complete sample details are provided in Table 1. Sample collection and processing were conducted in full compliance with international regulations, including the Nagoya Protocol, CITES, and relevant phytosanitary requirements.

**TABLE 1:** The information of samples.

Species	Purchasing company	Purchasing time	Lot no.	Sample no.
*Rubia cordifolia* L	Anguo City Juyaotang pharmaceutical Co., LTD.	2022/12/23	220906	Q_1
	Anguo City Juyaotang pharmaceutical Co., LTD.	2022/12/23	220907	Q_2
	Anguo City Juyaotang pharmaceutical Co., LTD.	2022/12/23	220915	Q_3
*Rubia yunnanensis* Diels	Kunming Chinese Medicine Factory limited	2022/12/27	220823	X_1
	Kunming Chinese Medicine Factory limited	2022/12/27	220905	X_2
	Kunming Chinese Medicine Factory limited	2022/12/27	220810	X_3
*Rubia wallichiana* Decne	Ningxia Multi-dimension Pharmaceutical Co., LTD.	2022/11/15	220924	Z_1
	Tibet Tibetan Medicine College Tibetan Medicine Co., LTD.	2022/12/19	2209211	Z_2
	Ritong Tibetan Medicine Factory	2022/12/21	2210034	Z_3
*Rubia schumanniana* Pritz	Sichuan new lotus herbal slices Co., LTD.	2022/11/25	2209121	DY_1
	Sichuan new lotus herbal slices Co., LTD.	2022/11/25	2209147	DY_2
	Sichuan new lotus herbal slices Co., LTD.	2022/11/25	2209183	DY_3
*Rubia magna* P. G. Xiao	Hekang Chinese Herbal Medicine Co., LTD.	2022/11/25	2209269	D_1
	Hekang Chinese Herbal Medicine Co., LTD.	2022/11/25	2209101	D_2
	Hekang Chinese Herbal Medicine Co., LTD.	2022/11/25	2209163	D_3

### 2.2 Instrumentation

Metabolomic analysis was conducted using a Thermo Fisher Vanquish ultra-high performance liquid chromatograph system coupled with an Orbitrap Exploris 120 mass spectrometer (Thermo Fisher Scientific, Waltham, MA, United States). For processing of the samples, a Heraeus Fresco 17 centrifuge (Thermo Fisher Scientific, Waltham, United States), a BSA124S-CW analytical balance (Sartorius, Gottingen, Germany), a PS-60AL ultrasonic instrument (Shenzhen Redbond Electronics Co., Ltd., Shenzhen, China), a JXFSTPRP-24 homogenizer (Shanghai Jingxin Technology Co., LTD., Shanghai, China), and a LGJ-10C freeze dryer (Sihuan Fricke instrument Technology Development Co., LTD., Beijing, China) were employed.

### 2.3 Metabolites extraction method

Each sample (100.0 mg ± 4.0 mg) was weighed and subjected to lyophilization. The samples were then mixed with 500 μL of extraction solution composed of methanol, acetonitrile, and water in a volume ratio of 2:2:1. The mixture was first vortexed for 30 s, then homogenized at 45 Hz for 4 min, followed by sonication in an ice-water bath (4°C) for 1 h. Subsequently, the samples were incubated at −20°C for 1 h. Centrifugation was conducted at 12,000 rpm (RCF = 13,800 × g, R = 8.6 cm) for 15 min at 4°C. The supernatant was carefully transferred to pre-cleaned glass vials for further analysis. For quality control (QC), a pooled QC sample was prepared by mixing equal volumes of all individual supernatants (Cai et al., 2024).

### 2.4 UPLC-MS/MS analysis

The UPLC-MS/MS analyses were conducted using a UHPLC system (Vanquish, Thermo Fisher Scientific) equipped with a Phenomenex Kinetex C18 column (2.1 mm × 100 mm, 2.6 μm) and interfaced with an Orbitrap Exploris 120 mass spectrometer (Thermo Fisher Scientific). The mobile phase A consisted of 0.01% acetic acid in water, while mobile phase B was a 1:1 (v/v) mixture of 2-propanol and acetonitrile. The auto-sampler was maintained at 4°C, and a 2 μL aliquot was injected for analysis.

The Orbitrap Exploris 120 mass spectrometer operated in information-dependent acquisition (IDA) mode, managed by Xcalibur software (Thermo Fisher Scientific). In this mode, the software continuously monitors the full scan MS spectrum. The conditions for the ESI source were set as follows: sheath gas flow rate of 50 Arb, auxiliary gas flow rate of 15 Arb, capillary temperature at 320°C, full MS resolution set at 60,000, MS/MS resolution at 15,000, collision energy utilizing stepped normalized collision energy (SNCE) values of 20/30/40, and a spray voltage of 3.8 kV for positive ion mode or −3.4 kV for negative ion mode (Hua et al., 2022).

### 2.5 Data preprocessing and statistical analysis

The raw data were converted to mzXML format using ProteoWizard and subsequently processed with a custom-built program designed in R (version 3.6.2), leveraging the XCMS package for peak detection, extraction, alignment, and integration. The XCMS parameters were optimized as follows: centWave algorithm, mass tolerance of 10 ppm, peak width range of 5–20 s, and a minimum signal-to-noise ratio (S/N) threshold of 3. For metabolite identification, we utilized the Biotree TCM standard product library (Biotree DB, version 3.0) along with public library BT-HERB. Within MetaboAnalyst 5.0 (https://www.metaboanalyst.ca/faces/ModuleView.xhtml), the UPLC-MS/MS data matrix underwent pretreatment involving relative standard deviation, logarithmic transformation, and pareto scaling (Bao et al., 2022). The standardization of data in both positive and negative ion modes was based on quality control (QC) samples. The areas of the detected peaks were used as input variables for subsequent analyses. Multivariate statistical analyses, including principal component analysis (PCA), orthogonal projections to latent structures-discriminant analysis (OPLS-DA), and hierarchical cluster analysis (HCA) were conducted using SIMCA-P software (version 13.0, Umetrics AB, Umea, Sweden). Different metabolites were selected based on variable importance in projection (VIP ≥1) and statistical significance (p-value ≤0.05, derived from Student’s t-test) (Hua et al., 2022). The identified metabolites were further annotated using the Kyoto Encyclopedia of Genes and Genomes (KEGG) pathway database to elucidate pathway associations (Cao et al., 2023). KEGG analysis was facilitated through the OmicShare tools, an online platform for data analysis (https://www.omicshare.com/tools).

## 3 Results

### 3.1 Metabolic profiles of Qiancao from different *Rubia* species

The analytical process commenced with a quality control (QC) evaluation of the UPLC-MS system performance. As shown in Supplementary Figure S1, the overlay of the total ion chromatograms (TICs) from QC samples demonstrated excellent retention time and peak intensity reproducibility. The high degree of chromatographic alignment, combined with inter-QC sample correlation coefficients >0.900, confirmed system stability throughout the analytical sequence. These results validate the instrumental stability and reliability of our metabolomics platform, thereby supporting the suitability of the methodology for subsequent large-scale metabolite profiling.

**FIGURE 1:**
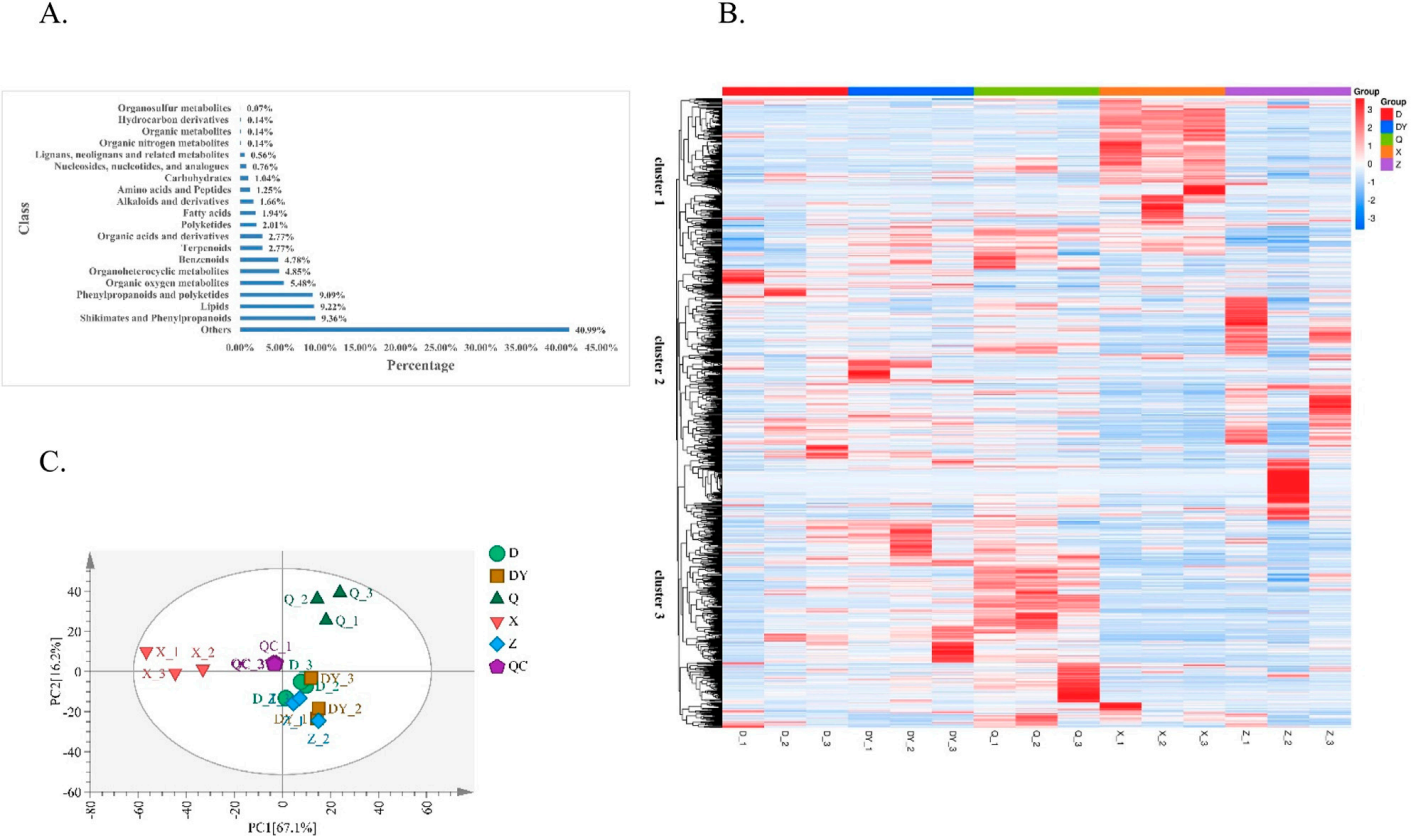
Metabolite profiles and multivariate analysis of Qiancao from different species (**(A)** Classification and proportion of metabolites; **(B)** Heatmap of the HCA; **(C)** PCA loading plot).

To systematically characterize the metabolite profiles across different Qiancao species, we conducted a metabolomics analysis of 15 independent sample batches. This comprehensive approach identified 1,440 metabolites, which encompass various categories, including shikimates and phenylpropanoids, lipids, phenylpropanoids and polyketides, organic oxygen metabolites, organoheterocyclic metabolites, benzenoids, terpenoids, organic acids and their derivatives, polyketides, fatty acids, alkaloids and their derivatives, amino acids and peptides, carbohydrates, nucleosides, nucleotides, and their analogues, lignans, neolignans and related metabolites, organic nitrogen metabolites, various organic metabolites, and hydrocarbon derivatives (Supplementary Table S1). Notably, the identified metabolites were predominantly composed of shikimates, phenylpropanoids, polyketides, lipids, as well as organic oxygen metabolites. The distribution percentages of the remaining identified metabolites are depicted in Figure 1A. Among these metabolites, there are essential primary metabolites critical for plant growth and development, including carbohydrates, fatty acids, nucleosides, nucleotides, and amino acids, etc., These metabolites primarily provide energy for medicinal plants, contribute to cell structure, and facilitate growth and development. Furthermore, we detected numerous secondary metabolites (active metabolites), such as alkaloids, lignans, neolignans, phenols, flavonoids, terpenoids, organic acids, phenylpropanoids, and polyketides. Typically, these substances serve as defense mechanisms against pests and diseases or enable adaptation to their environment, often possessing potential medicinal value.

### 3.2 Identification of differences among the metabolite profiles by multivariate analysis

The differentially abundant metabolites were normalized and used to generate a clustering heatmap, which visually represents the species-specific regulatory mechanisms underlying metabolite variations in Qiancao species (Figure 1B). hierarchical cluster analysis (HCA) clearly segregated the five Qiancao species into distinct groups, with the metabolites forming three major clusters: cluster 1 was characterized by metabolites that peaked in species X, cluster 2 encompassed metabolites that were most abundant in species D, DY, Q, and Z, while cluster 3 contained metabolites that reached their highest levels in species DY and Q. This clustering pattern demonstrates the considerable diversity in metabolite profiles across various Qiancao species, implying that their pharmacological effects may also differ significantly. Additionally, the proximity of biological replicates in the clustering indicates a high degree of consistency and reliability in the biological data.

Principal component analysis (PCA) was performed to reduce data dimensionality and reveal the underlying structure of multiple variables through principal component extraction. In the PCA score plot (Figure 1C), the quality control (QC) samples - prepared as pooled extracts from all samples - clustered tightly, demonstrating the analytical system’s stability and reproducibility. The first two principal components (PC1 and PC2) accounted for 67.1% and 16.2% of the total variance, respectively. The PCA results revealed clear sample segregation along the principal components, with distinct species-specific clustering patterns. Notably, there was a distinct separation between the Q and X samples and the others, while the DY, D, and Z samples were grouped together, showing minimal separation.

In summary, both hierarchical cluster analysis (HCA) and principal component analysis (PCA) revealed significant metabolic disparities among the Qiancao samples, particularly between samples Q and X, which showed the most pronounced variations. However, it should be noted that PCA, as an unsupervised multivariate technique, has limitations in detecting intra-group variability and random errors that may not align with the research objectives. Furthermore, PCA’s inability to account for complex details and its tendency to overlook overarching trends reduce its effectiveness in highlighting intergroup differences or in identifying specific differential metabolites (Hua et al., 2022). Given these intrinsic constraints, the PCA model struggled to accurately differentiate the metabolites present in the five distinct Qiancao samples. In contrast, the orthogonal partial least squares discriminant analysis (OPLS-DA), a supervised multivariate approach, demonstrated superior performance by: (1) effectively filtering out system noise and (2) amplifying biologically relevant metabolic signatures (Bao et al., 2022). Thus, integration of complementary statistical approaches is recommended to obtain more accurate and comprehensive metabolic insights.

### 3.3 Differentially accumulated metabolites screening

**FIGURE 2:**
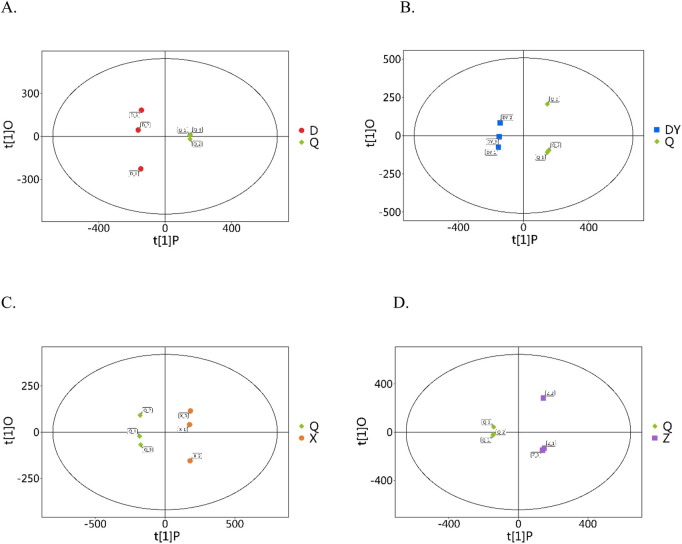
OPLS-DA score plot (**(A)** Q vs. D; **(B)** Q vs. DY; **(C)** Q vs. X; **(D)** Q vs. Z).

Orthogonal partial least squares discriminant analysis (OPLS-DA) integrates orthogonal signal correction (OSC) and partial least squares discriminant analysis (PLS-DA) to effectively remove extraneous variations while enhancing detection of biologically relevant discriminatory variables (Cao et al., 2023). To efficiently classify two species samples, we employed OPLS-DA to develop discrimination models, with species sources serving as the grouping variables. It is important to note that, aside from the Q sample, the other species are not recognized in the most recent edition of the National Pharmacopeia of China. Therefore, we focused on identifying differential metabolites between Q and the non-pharmacopeial species. In the OPLS-DA modeling, R2X and R2Y represent the explanatory power of the X and Y matrices, respectively, while Q2 signifies the model’s predictive capability. The R2X, R2Y, and Q2 values for the OPLS-DA models exceeded 0.7, 0.9, and 0.8, respectively (Supplementary Table S2), indicating the models’ robustness and reliability. Score plot visualization of OPLS-DA clearly illustrated that Q samples were distinctly differentiated from the other species (D, DY, X, and Z) (Figure 2). To assess whether the model was susceptible to overfitting, we conducted 200 permutation tests. The results indicated that all R2 and Q2 values derived from the random permutations were lower than the original values (Supplementary Figure S2), thereby confirming the model’s robustness, reproducibility, and absence of overfitting. The predictive performance remained within acceptable limits.

**FIGURE 3:**
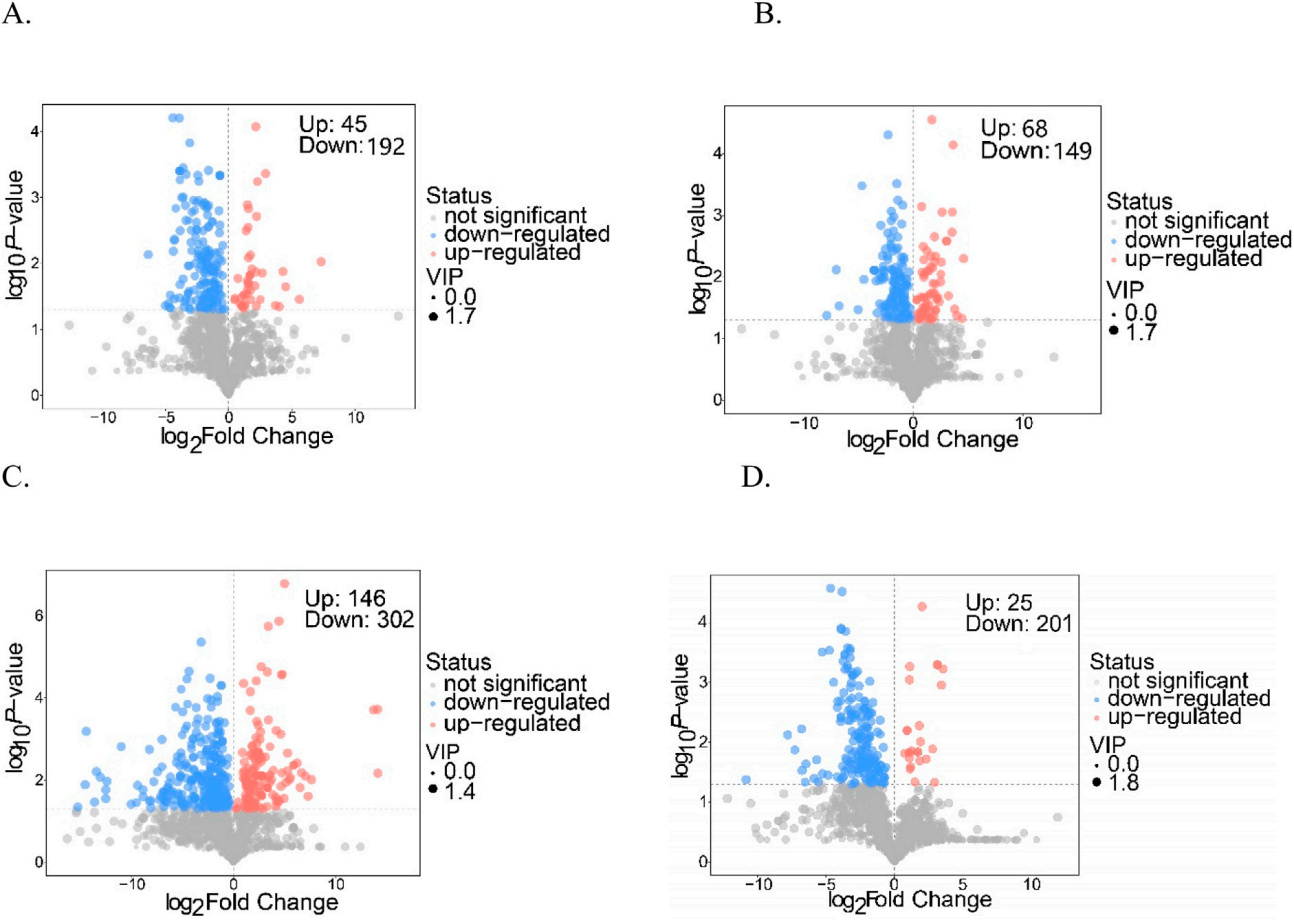
Volcano plots of each group (**(A)** Q vs. D; **(B)** Q vs. DY; **(C)** Q vs. X; **(D)** Q vs. Z).

Differentially accumulating metabolites (DAMs) were selected according to fold change values (FC ≥2 or ≤0.5), with further identification achieved through their variable importance in projection scores (VIP >1). These screening results are visualized in the volcano plots shown in Figure 3. Notably, the comparative analysis of the Q and D groups revealed a total of 237 metabolites exhibiting differential accumulation, which included 192 downregulated and 45 upregulated metabolites (Figure 3A). Similarly, the comparison between the Q and DY groups identified 217 differentially accumulated metabolites, comprising 149 that were downregulated and 68 that were upregulated (Figure 3B). The Q versus X group comparison yielded the largest number of differentially accumulated metabolites, totaling 448, with 302 downregulated and 146 upregulated (Figure 3C). Finally, the Q versus Z group comparison highlighted 226 differentially accumulated metabolites, consisting of 201 downregulated and 25 upregulated metabolites (Figure 3D). The Q vs. X comparison showed the most substantial metabolic differences, with nearly double the number of differential metabolites compared to other groups, highlighting significant metabolic divergence between these two species. Furthermore, the major categories of differentially accumulated metabolites across all four comparison groups include phenylpropanoids, shikimates, polyketides, lipids, and organic metabolites, as outlined in Supplementary Table S3. These metabolite classes were identified as the key factors contributing to the observed disparities among the samples.

K-means clustering analysis classified the differentially accumulated metabolites into nine distinct subclasses (Supplementary Figure S3), with each cluster exhibiting unique accumulation patterns: cluster 1 included 82 metabolites significantly upregulated in DY, cluster 2 comprised 60 metabolites predominantly elevated in D, cluster 3 contained 43 metabolites enriched in DY, cluster 4 encompassed 112 metabolites that demonstrated marked accumulation in Q, cluster 5 featured 40 metabolites upregulated in both Q and X, cluster 6 included 63 metabolites characteristically increased in Z, cluster 7 contained 78 metabolites also upregulated in Z, cluster 8 comprised 148 metabolites exhibiting upregulation in X, and cluster 9 included 31 metabolites specifically accumulated in Q. To further characterize these metabolic differences, we performed a matchstick plot analysis to highlight the most significantly differential metabolites across all four comparison groups (Supplementary Figure S4; Table 2). Collectively, these results demonstrate substantial interspecies variation in both the abundance and distribution patterns of differential metabolites among the Qiancao samples.

**TABLE 2:** Top 20 of the significantly different metabolites in 4 comparison groups.

Group	Name	VIP	Fold change	Log_ fold change	Type
Q vs. D	Linderane	1.68	4.42	2.14	up-regulated
	10-deacetylbaccatin III	1.65	7.57	2.92	up-regulated
	Isorhamnetin 3-galactoside	1.49	4.84	2.28	up-regulated
	6-[(2R)-2,3-dihydroxy-3-methyl-butyl]-7-hydroxy-chromen-2-one	1.67	2.82	1.49	up-regulated
	Santonin	1.68	2.95	1.56	up-regulated
	Isoflavone base + 2O, O-Hex	1.66	4.62	2.21	up-regulated
	aschantin	1.66	2.90	1.53	up-regulated
	Ethyl caffeate	1.65	2.61	1.38	up-regulated
	Citrusinine_I	1.55	3.30	1.72	up-regulated
	Dimethyl (R)-(+)-malate	1.60	2.97	1.57	up-regulated
	Puerarin	1.68	0.05	−4.41	down-regulated
	gamma-Decalactone	1.61	0.07	−3.90	down-regulated
	9-(2,3-dihydroxy-3-methylbutoxy)-4-methoxyfuro[3,2-g] chromen-7-one	1.51	0.12	−3.08	down-regulated
	5-[(2R,3S)-6-hydroxy-2-(4-hydroxyphenyl)-4-[(E)-2-(4-hydroxyphenyl) ethenyl] −2,3-dihydro-1-benzofuran-3-yl] benzene −1,3-diol	1.58	0.01	−6.10	downregulated
	1,7-Dihydroxyxanthone	1.68	0.08	−3.60	down-regulated
	Phenethyl sophoroside	1.68	0.33	−1.59	down-regulated
	[(1S)-2-hydroxy-1-[(R)-hydroxy-(7-methoxy-2-oxo-chromen-6-yl) methyl]-2-methyl-propyl] (Z)-2-methylbut-2-enoate	1.67	0.07	−3.87	down-regulated
	[2-hydroxy-1-[hydroxy-(7-methoxy-2-oxo-chromen-6-yl) methyl]-2-methyl-propyl] (E)-2-methylbut-2-enoate	1.67	0.07	−3.87	down-regulated
	[2-hydroxy-1-[hydroxy-(7-methoxy-2-oxo-chromen-6-yl) methyl]-2-methyl-propyl] (Z)-2-methylbut-2-enoate	1.67	0.07	−3.87	down-regulated
	[2,3-dihydroxy-1-(7-methoxy-2-oxo-chromen-6-yl)-3-methyl-butyl] (Z)-2-methylbut-2-enoate	1.67	0.07	−3.87	down-regulated
Q vs. DY	Loganin	1.71	3.30	1.72	up-regulated
	(2S)-6-[(2S)-5,7-dihydroxy-2-(4-hydroxyphenyl)-4-oxo-2,3-dihydrochromen-8-yl]-5,7-dihydroxy-2-(4-hydroxyphenyl)-2,3-dihydrochromen-4-one	1.51	12.61	3.66	up-regulated
	CARMINIC_ACID	1.65	1.73	0.79	up-regulated
	Chalcomoracin	1.51	11.96	3.58	up-regulated
	10-deacetylbaccatin III	1.65	6.28	2.65	up-regulated
	3-(2,4-dihydroxyphenyl)-7-hydroxy-6,8-bis(3-methylbut-2-enyl)-2,3-dihydrochromen-4-one	1.66	11.76	3.56	up-regulated
	N1, N5, N10, N14-Tetra-trans-p-coumaroylspermine	1.65	3.90	1.96	up-regulated
	Raffinose	1.66	8.32	3.06	up-regulated
	Manninotriose	1.66	8.32	3.06	up-regulated
	gamma-Decalactone	1.70	0.10	−3.38	down-regulated
	Alizarin	1.68	0.21	−2.26	down-regulated
	5-[(2R,3S)-6-hydroxy-2-(4-hydroxyphenyl)-4-[(E)-2-(4-hydroxyphenyl) ethenyl]-2,3-dihydro-1-benzofuran-3-yl] benzene-1,3-diol	1.45	0.04	−4.63	down-regulated
	Isosinensetin	1.69	0.39	−1.37	down-regulated
	7-hydroxy-3-phenyl-chromen-4-one	1.67	0.53	−0.93	down-regulated
	Benzoylpaeoniflorin	1.59	0.26	−1.96	down-regulated
	Riboflavin	1.67	0.30	−1.75	down-regulated
	Androsin	1.67	0.26	−1.97	down-regulated
	(2E,4E)-Hexa-2,4-dienoic acid	1.62	0.49	−1.02	down-regulated
Q vs. X	[(1S)-5-hydroxy-1-[(2S,3R,4S,5S,6R)-3,4,5-trihydroxy-6-(hydroxymethyl) oxan-2-yl] oxy-1,4a,5,7a-tetrahydrocyclopenta[c]pyran-7-yl] methyl benzoate	1.41	32.25	5.01	up-regulated
	valeramide	1.42	22.25	4.48	up-regulated
	1,4-naphthalene-dione	1.41	10.74	3.43	up-regulated
	Phenylethyl 2-Glucoside	1.42	6.69	2.74	up-regulated
	Protogenkwanin 4′-glucoside	1.42	10.09	3.33	up-regulated
	Alisol B Acetate	1.38	27.80	4.80	up-regulated
	Soyasapogenol A	1.39	26.27	4.72	up-regulated
	Tyromycic_acid	1.42	4.74	2.24	up-regulated
	Loganin	1.42	2.01	1.01	up-regulated
	Androsin	1.41	3.19	1.68	up-regulated
	Alizarin	1.43	0.11	−3.15	down-regulated
	8-O-4-Hydroxycinnamoylharpagide	1.40	0.05	−4.33	down-regulated
	(2E,4E)-Hexa-2,4-dienoic acid	1.42	0.21	−2.27	down-regulated
	13,15-dihydroxy-5-methyl-4-oxabicyclo [10.4.0] hexadeca-1(12),13,15-triene-3,11-dione	1.31	0.04	−4.53	down-regulated
	(E)-9,12,13-trihydroxyoctadec-10-enoic acid	1.42	0.44	−1.18	down-regulated
	(E,9S,12S,13S)-9,12,13-trihydroxyoctadec-10-enoic acid	1.42	0.44	−1.18	down-regulated
	columbianetin	1.09	0.03	−5.11	down-regulated
	7-hydroxy-3-phenyl-chromen-4-one	1.42	0.34	−1.55	down-regulated
	Mulberroside A	1.37	0.00	−9.81	down-regulated
	quinine	1.40	0.18	−2.51	down-regulated
Q vs. Z	Podophyllotoxin	1.76	4.01	2.00	up-regulated
	Raffinose	1.74	8.65	3.11	up-regulated
	Manninotriose	1.74	8.65	3.11	up-regulated
	Ethyl Orsellinate	1.72	2.13	1.09	up-regulated
	(2R,3R)-3,5-dihydroxy-2-(4-hydroxyphenyl)-8-(3-methylbut-2-enyl)-7-[(2S,3R,4S,5S,6R)-3,4,5-trihydroxy-6-(hydroxymethyl) oxan-2-yl] oxy-2,3-dihydrochromen-4-one	1.55	11.59	3.53	up-regulated
	Osajin	1.71	2.11	1.08	up-regulated
	Gentianose	1.69	10.67	3.42	up-regulated
	Hexamethylquercetagetin	1.70	3.48	1.80	up-regulated
	2-Hydroxy-3-(4-hydroxyphenyl) propanoic acid	1.67	1.87	0.90	up-regulated
	2,3-Dihydroxy-4-methoxyacetophenone	1.67	1.87	0.90	up-regulated
	9-(2,3-dihydroxy-3-methylbutoxy)-4-methoxyfuro[3,2-g] chromen-7-one	1.74	0.04	−4.68	down-regulated
	8-O-4-Hydroxycinnamoylharpagide	1.75	0.07	−3.85	down-regulated
	Alizarin	1.67	0.12	−3.11	down-regulated
	Isoleucine	1.73	0.07	−3.92	down-regulated
	Piperidine	1.74	0.07	−3.90	down-regulated
	Puerarin	1.70	0.08	−3.57	down-regulated
	Isoimperatorin	1.63	0.09	−3.45	down-regulated
	1,7-Dihydroxyxanthone	1.62	0.04	−4.78	down-regulated
	Mukonine	1.58	0.10	−3.27	down-regulated

### 3.4 Enrichment and pathway analyses of the differentially accumulated metabolites

**FIGURE 4:**
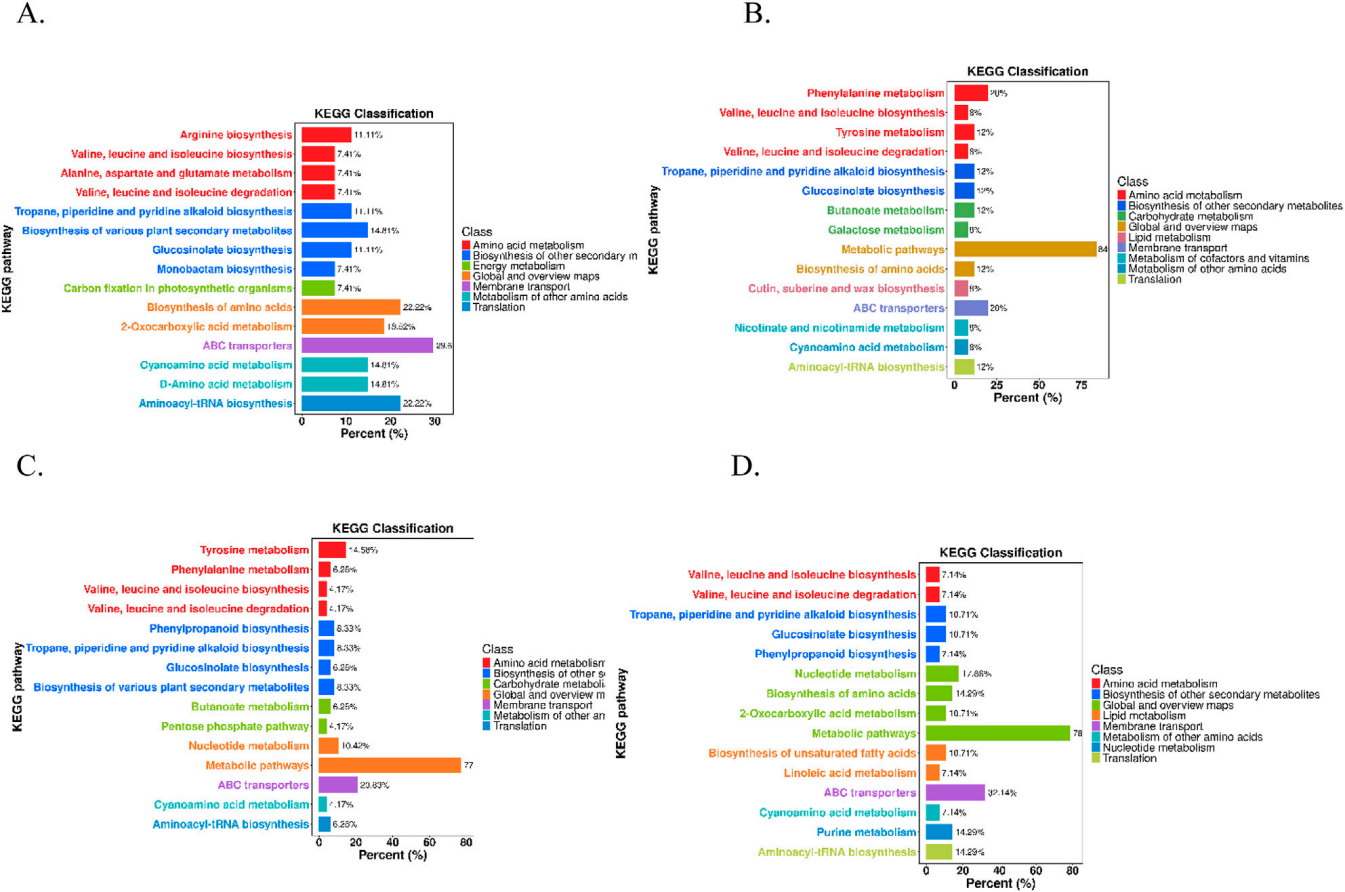
KEGG classification maps of the differentially accumulated metabolites (**(A)** Q vs. D; **(B)** Q vs. DY; **(C)** Q vs. X; **(D)** Q vs. Z).

The *in vivo* interactions of differentially accumulated metabolites play a crucial role in the development of unique metabolic pathways. The identification of these metabolites across various comparison groups was accomplished through annotation with the Kyoto Encyclopedia of Genes and Genomes (KEGG) database. The subsequent analysis of these annotations for enrichment revealed pathways with a significant abundance of differentially accumulated metabolites (Figure 4). In the comparison between the Q and D groups, notable enrichment was observed primarily in pathways associated with ABC transporters, aminoacyl-tRNA biosynthesis, amino acid biosynthesis, and 2-oxocarboxylic acid metabolism. In the Q vs. DY comparison groups, enriched pathways included metabolic pathways, phenylalanine metabolism, and ABC transporters. In the Q vs. X groups, the prominent enriched pathways were metabolic pathways, ABC transporters, and tyrosine metabolism. Conversely, in the Q vs. Z comparisons, significant enrichment was primarily observed in metabolic pathways, ABC transporters, and nucleotide metabolism. Notably, several pathways, including metabolic pathways, ABC transporters, and amino acid metabolism, were consistently enriched across multiple comparisons, highlighting their fundamental roles in interspecies metabolic variation. These enriched pathways have distinct biological significance. Metabolic pathways are crucial to primary metabolism, facilitating essential processes that support plant growth, development, and reproduction. They provide both precursors and energy for the biosynthesis of secondary metabolites, which are fundamental in plant defense strategies, signaling processes, and adaptation to environmental stresses (Erb and Kliebenstein, 2020). ABC transporters constitute a large family of proteins that harness energy from ATP hydrolysis to transport a variety of molecules across cellular membranes. They are critical for the distribution and accumulation of secondary metabolites within plant tissues (Geisler, 2024). Moreover, the metabolism of amino acids is essential for the production of precursors and the provision of critical building blocks for a diverse range of secondary metabolites.

**FIGURE 5:**
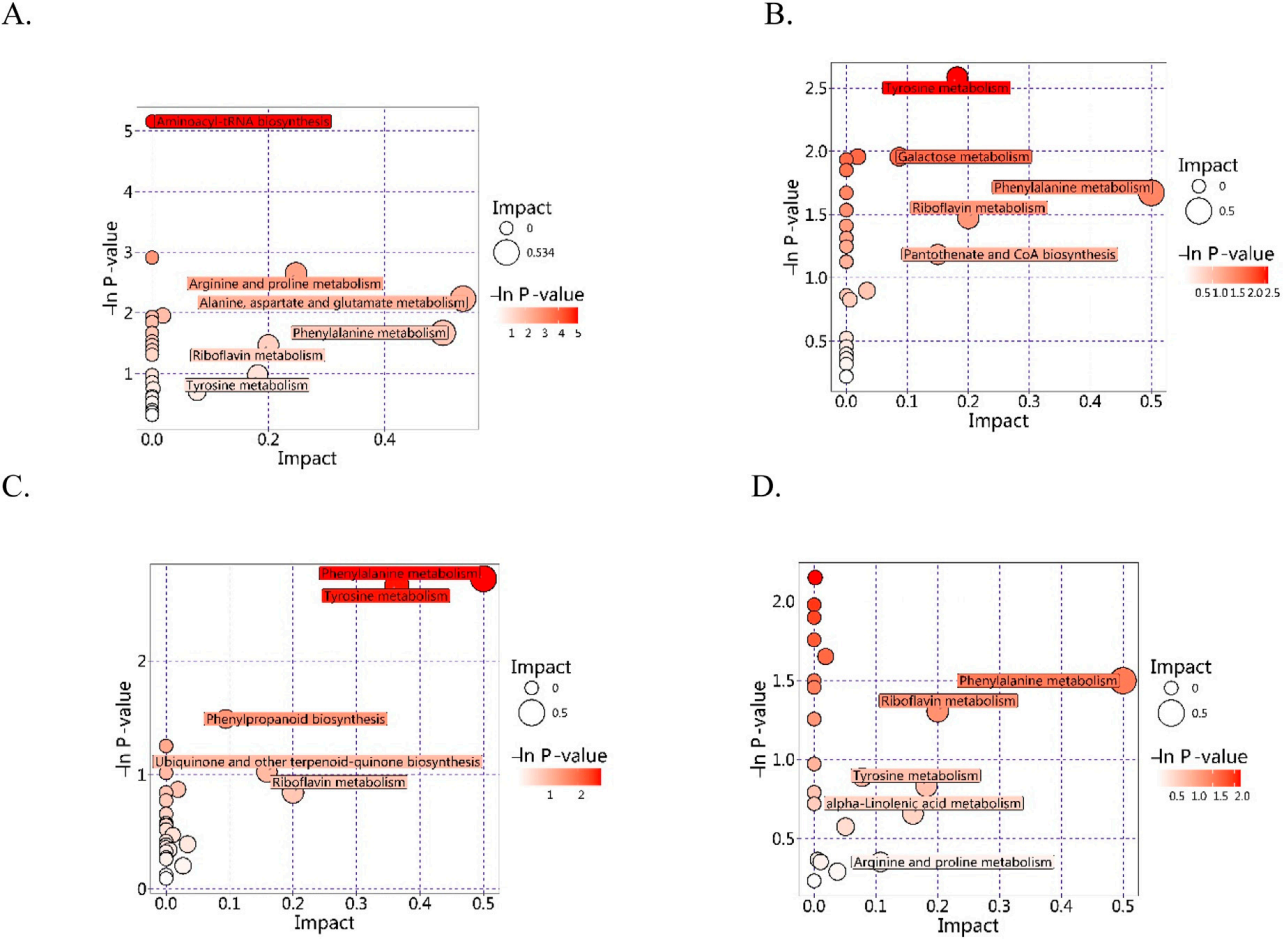
KEGG pathway bubble plot (**(A)** Q vs. D; **(B)** Q vs. DY; **(C)** Q vs. X; **(D)** Q vs. Z).

Our pathway enrichment analysis revealed a notable enrichment in metabolic pathways. To elucidate the specific pathways involved, we analyzed the differentially accumulated metabolites between species Q and the other species using the KEGG database. This analysis identified eight potential target metabolic pathways with pathway impact scores exceeding 0.1, as presented in Table 3 and illustrated in Figure 5. The pathways identified include metabolism of alanine, aspartate, and glutamate; metabolism of arginine and proline; biosynthesis of pantothenate and CoA; biosynthesis of ubiquinone and other terpenoid-quinones; metabolism of phenylalanine; metabolism of riboflavin; metabolism of tyrosine; and metabolism of alpha-linolenic acid. Notably, the metabolic pathways targeted in Qiancao exhibited variation among different species, with amino acid metabolism, riboflavin metabolism, and terpenoid metabolism being most prominently represented. Within these pathways, several specific metabolites were identified, including fumaric acid, L-arginine, L-glutamic acid, tyramine, 4-hydroxycinnamic acid, homogentisic acid, L-aspartic acid, and L-phenylalanine, all linked to amino acid metabolism. Riboflavin played a role in riboflavin metabolism, while 4-hydroxycinnamic acid and homogentisic acid contributed to terpenoid biosynthesis. These metabolites exhibit distinct functions within their respective metabolic pathways, for instance, L-glutamic acid is crucial to amino acid metabolism, acting as an amino group donor in transamination reactions that are vital for amino acid synthesis (Liao et al., 2022). Fumaric acid, integral to both the urea cycle and the citric acid cycle, indirectly supporting amino acid biosynthesis by contributing intermediates to these metabolic pathways (Zhang et al., 2023). Riboflavin plays a pivotal role in its own metabolism, as its conversion into flavin mononucleotide and flavin adenine dinucleotide is essential for various enzymatic processes that facilitate cellular energy generation, fatty acid oxidation, amino acid metabolism, and the mechanisms of antioxidant defense (Chengalroyen et al., 2024). While 4-hydroxycinnamic acid and homogentisic acid are primarily involved in phenylpropanoid and amino acid catabolic pathways, their metabolic networks may indirectly influence terpenoid metabolism through shared precursors or regulatory mechanisms.

**TABLE 3:** Pathway analysis results of different Qiancao.

Group	Pathway	Raw p	Holm adjust	FDR	Impact	Pathway metabolites
Q vs. D	Arginine and proline metabolism	0.07	1.00	1.00	0.25	L-Aspartic acid; L-Arginine; L-Glutamic acid
	Alanine, aspartate and glutamate metabolism	0.11	1.00	1.00	0.53	L-Aspartic acid; L-Glutamic acid
	Phenylalanine metabolism	0.19	1.00	1.00	0.50	L-Phenylalanine
	Riboflavin metabolism	0.23	1.00	1.00	0.20	Riboflavin
	Tyrosine metabolism	0.38	1.00	1.00	0.18	Tyramine
Q vs. DY	Tyrosine metabolism	0.08	1.00	1.00	0.18	Tyramine; Fumaric acid
	Phenylalanine metabolism	0.19	1.00	1.00	0.50	L-Phenylalanine
	Riboflavin metabolism	0.23	1.00	1.00	0.20	Riboflavin
	Pantothenate and CoA biosynthesis	0.31	1.00	1.00	0.15	Pantothenic acid
Q vs. X	Phenylalanine metabolism	0.07	1.00	1.00	0.50	L-Phenylalanine; 4-Hydroxycinnamic acid
	Tyrosine metabolism	0.07	1.00	1.00	0.36	Homogentisic acid; Tyramine; Fumaric acid
	Ubiquinone and other terpenoid-quinone biosynthesis	0.36	1.00	1.00	0.16	4-Hydroxycinnamic; Homogentisic acid
	Riboflavin metabolism	0.43	1.00	1.00	0.20	Riboflavin
Q vs. Z	Phenylalanine metabolism	0.22	1.00	1.00	0.50	L-Phenylalanine
	Riboflavin metabolism	0.27	1.00	1.00	0.20	Riboflavin
	Tyrosine metabolism	0.44	1.00	1.00	0.18	Tyramine
	alpha-Linolenic acid metabolism	0.52	1.00	1.00	0.16	Alpha-Linolenic acid
	Arginine and proline metabolism	0.70	1.00	1.00	0.11	L-Arginine

(All pathways shown in the table are potential target metabolic pathways with pathway impacts of above 0.1; Pathway metabolites, the actually matched metabolite in the pathway; Holm adjust, *p* value adjusted by Holm-Bonferroni method; FDR, p value adjusted using False Discovery Rate; Impact, pathway impact value.).

## 4 Discussion

The *Rubiaceae* family comprises a wide variety of plants that have been employed for centuries in traditional medicine, dye manufacturing, and various other applications. Within this family, several species of the genus *Rubia* are particularly noteworthy. Notably, species Q (*Rubia cordifolia* L.) has received the most extensive research attention and practical application. Other congeners including species DY, D, X, and Z exhibit comparable metabolites and therapeutic efficacy, positioning them as potential alternatives to species Q. These species hold significant potential as valuable resources for medicinal use. To thoroughly investigate their potential as alternatives, we conducted a comprehensive metabolomic analysis comparing the secondary metabolites of species Q with those of the other species, as these specialized metabolites largely determine their pharmacological activities. Current research has identified several secondary metabolites as key contributors to Qiancao’s antitumor properties. Particularly noteworthy are alizarin and mollugin, which exhibit significant anti-proliferative effects against cancer cells *in vitro*, highlighting their therapeutic potential for oncology applications. Additionally, rubiadin is recognized for its hepatoprotective properties, indicating its role in supporting liver health and protecting against liver toxicity (Shan et al., 2016). Furthermore, the polysaccharides obtained from Qiancao, along with loganin and mollugin, possess neuroprotective activity, as evidenced by their ability to mitigate neuronal damage and neuroinflammation (Wen et al., 2022). The presence of polyphenols and flavonoids metabolites in Qiancao, such as isorhamnetin 3-galactoside, aschantin and chalcomoracin, contributes to its antioxidant effects, which are critical in combating oxidative stress-related diseases (Jing et al., 2015; Zhou and Feng, 2023). Additionally, Qiancao contains antiviral compounds such as citrusinine I, hypoxanthine, xanthopurpurin, and vanillic acid, which have shown promising inhibitory effects against various viruses in preclinical studies (Sun et al., 2016). In summary, Qiancao possesses rich metabolites that supports its widespread traditional medicinal applications. However, different species of Qiancao contain varying metabolites, leading to the differences in their pharmacological activities. In this study, we employed UPLC-MS/MS to characterize the metabolomes of species Q, DY, D, X, and Z. Multivariate statistical analysis revealed significant variations among the species. Notably, the metabolic profiles of X and Z were distinctly different from those of species Q, while DY and D exhibited profiles that were similar to Q, albeit with notable differences. Principal component analysis (PCA) and orthogonal partial least squares discriminant analysis (OPLS-DA) effectively distinguished Q from the other species. However, ascertaining whether DY, D, X, and Z can be considered as alternatives to Q, and whether they should be included in the Pharmacopoeia of the People’s Republic of China, requires further investigation beyond mere metabolite analysis. A multidimensional evaluation of their pharmacological properties, toxicological profiles, and clinical efficacy is imperative. In our future research, we intend to carry out a series of pharmacological activity assessments to investigate the effects of various Qiancao extracts on specific targets, including certain enzymes and receptors. Concurrently, we will assess the ADMET (absorption, distribution, metabolism, excretion, and toxicity) properties of the selected Qiancao species. This evaluation will employ computer-aided drug design (CADD) techniques in conjunction with *in vitro* experiments to predict the interactions of metabolites and their potential toxicological profiles. Additionally, we will perform acute toxicity studies to assess the safety and toxicological manifestations associated with different Qiancao species. A comprehensive evaluation of the efficacy and toxicity differences among these species will be conducted to establish a scientific foundation for future clinical applications. Furthermore, investigating ecogeographical influences on metabolite biosynthesis will address sustainability considerations (Li et al., 2024). This comprehensive approach ensures that the potential substitutes are not only pharmacologically effective but also sustainable and environmentally adaptable. Moreover, integrating advanced biotechnological techniques, such as CRISPR-Cas9 for gene editing (Saini et al., 2023), could enhance the desirable traits in DY, D, X, and Z, making them even more viable as substitutes for Q. This comprehensive strategy facilitates a thorough and well-rounded assessment of the potential of these species. Additionally, it is vital to take socio-economic considerations into account, such as cultivation expenses and market acceptance. Collaborating with local communities that have traditionally utilized these plants can provide significant ethnobotanical knowledge. By merging scientific investigation with indigenous wisdom, we can create a more inclusive and culturally sensitive integration into modern medicine. Ultimately, developing standardization and quality control measures will be essential to guarantee consistency and safety across various Qiancao species. Through meticulous scientific validation and cooperative efforts, these species may be elevated to a status comparable to Q, thus broadening their applications in both traditional and modern therapeutic contexts.

Metabolic pathway analysis integrates metabolomic data with biological context, thereby mapping metabolite variations onto functionally annotated metabolic pathways (Zhou et al., 2024). The KEGG pathway analysis of differential metabolites conducted in this study revealed their significant participation in several metabolic pathways. These include alanine, aspartate, and glutamate metabolism; arginine and proline metabolism; biosynthesis of pantothenate and CoA; biosynthesis of ubiquinone and other terpenoid-quinone metabolites; as well as phenylalanine, riboflavin, tyrosine, and alpha-linolenic acid metabolism. Notably, these pathways collectively represent core components of amino acid metabolism networks. Additionally, specific metabolites within these critical pathways were identified, serving as a foundational reference for understanding the biosynthesis of secondary metabolites in plants and exploring the mechanisms that contribute to quality formation. Future studies should focus on conducting a comprehensive quantitative analysis of these differential metabolites to better understand the variations in pharmacodynamic metabolites among different species of Qiancao. Moreover, the specific enzymes, proteins, and genes that govern the biosynthetic pathways of these metabolites are still unknown. This highlights the need for in-depth investigations in genomics, proteomics, and transcriptomics to unveil the molecular mechanisms underlying metabolite production. It is essential to identify the genes and proteins involved in the synthesis of Qiancao metabolites. Additionally, establishing a regulatory network that connects genes, proteins, and metabolites is important. This can be achieved through precise quantitative analysis of metabolites and the overexpression of relevant genes and proteins, which will enhance our understanding of the molecular mechanisms of metabolite biosynthesis in various Qiancao species. Furthermore, employing multi-omics strategies, including metabolomics, transcriptomics, and proteomics, can provide a comprehensive view of the regulatory networks involved (Naik et al., 2023). The integration of these multi-omics datasets will elucidate the complex interactions among biological molecules, thereby identifying potential targets for Qiancao quality improvement through genetic or biochemical interventions. Such integrative approaches will not only deepen our understanding of the metabolic intricacies of Qiancao but also facilitate the development of innovative breeding techniques and metabolic engineering strategies. Future investigations should also account for the environmental influences on metabolite biosynthesis, which could lead to optimized cultivation methodologies. Additionally, collaborative efforts in bioinformatics and systems biology may assist in constructing predictive models that simulate the metabolic pathways in Qiancao. These models would enable us to forecast variations in metabolite flux under different conditions, thus helping identify key regulatory nodes and bottlenecks within the biosynthetic pathways. Ultimately, these predictive models, when combined with experimental validation, could revolutionize our methods for cultivating Qiancao and enhancing its metabolites. By harnessing advanced computational tools and high-throughput technologies, we can gain a deeper and more accurate understanding of plant metabolic networks. This, in turn, presents unprecedented opportunities to improve therapeutic potential and commercial applications of Qiancao.

## 5 Conclusion

In conclusion, this study employed the UPLC-MS/MS metabolomics technique to characterize metabolic differences among five species of Qiancao, specifically Q, D, DY, X, and Z. This analytical approach facilitated the detection of 1,440 metabolites. Specifically, we identified 237, 217, 448, and 226 differentially accumulated metabolites in the comparisons of Q versus D, Q versus DY, Q versus X, and Q versus Z, respectively. Multivariate statistical analyses confirmed significant interspecies metabolic variations. Pathway enrichment analysis highlighted the predominant involvement of these metabolites in three key pathways, including amino acid metabolism, riboflavin metabolism, and terpenoid biosynthesis. Notably, we also identified metabolites that varied across these pathways. The results of this study demonstrate that combining metabolomics with chemometric techniques is an effective method for identifying potential metabolites in different Qiancao species. These findings provide a solid foundation for future research into the identification, quality control, metabolite biosynthesis, and bioactivity evaluation of Qiancao.

## Data Availability

The original contributions presented in the study are included in the article/Supplementary Material, further inquiries can be directed to the corresponding author.
