# Supplementary material for: Qiancao (Rubia spp.) – a comprehensive metabolomics analysis of the five core species
Source: Front Pharmacol. 2025 Aug 18;16:1541994. doi: 10.3389/fphar.2025.1541994 (PMC12399549; doi:10.3389/fphar.2025.1541994)
Supplement: Supplementary file 1 [file Supplementaryfile1.docx]

Supplementary Material

# Supplementary Figures

A.


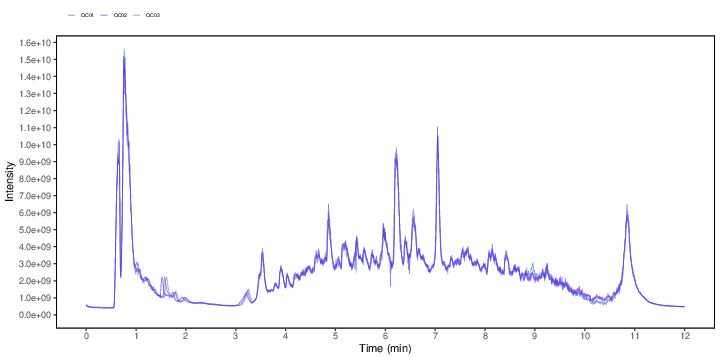


B.


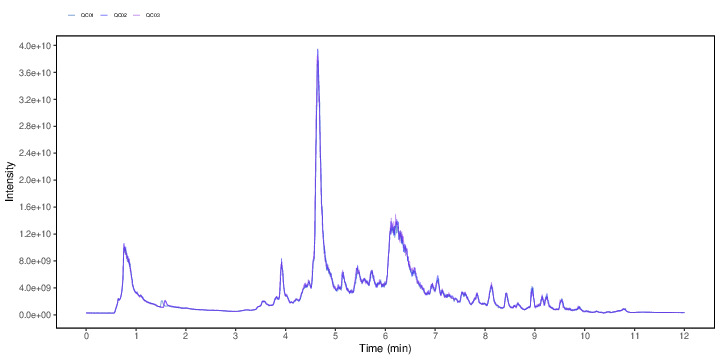


C.


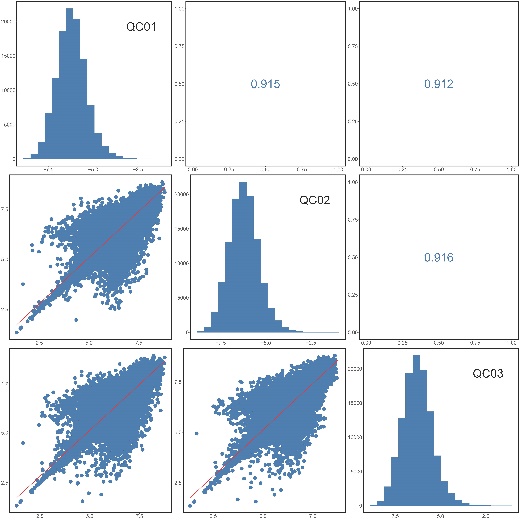


**Supplementary Figure 1** Quality control analysis (A. QC sample superposition in positive ion mode. B. QC sample superposition in negative ion mode. C. Correlation analysis results of QC sample.)

A. B.


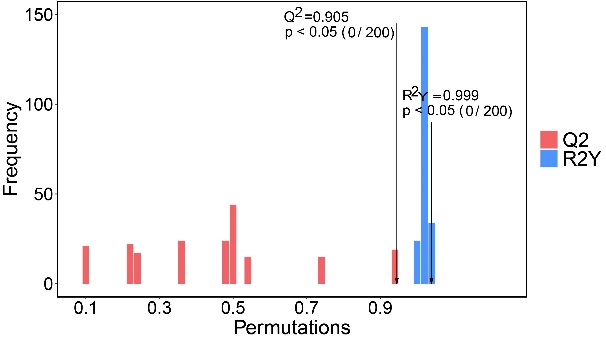

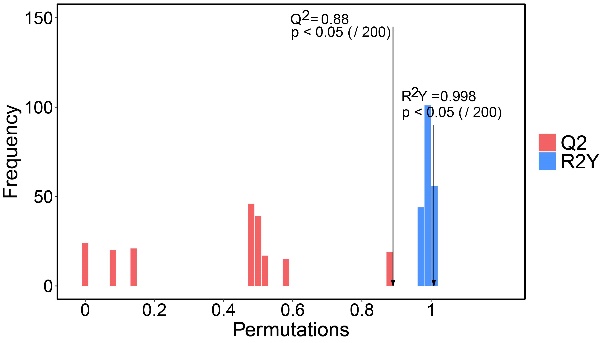


C. D.


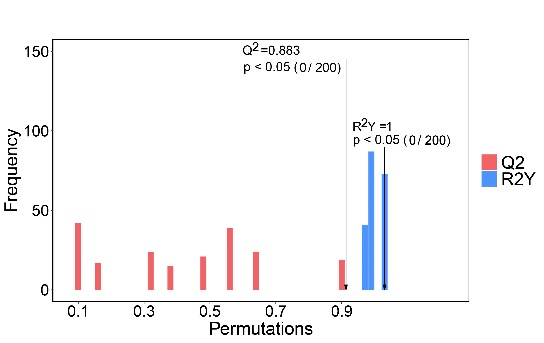

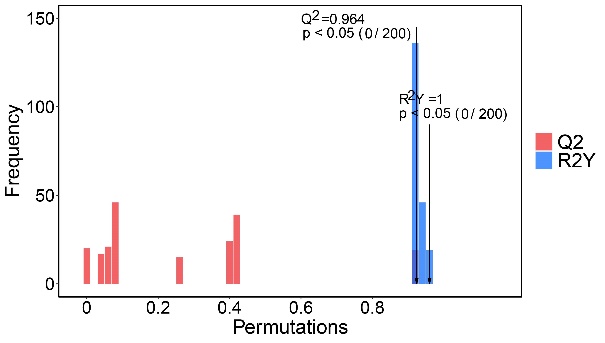


**Supplementary Figure 2** OPLS-DA permutation histograms (A. Q vs D; B. Q vs DY; C. Q vs X; D. Q vs Z)


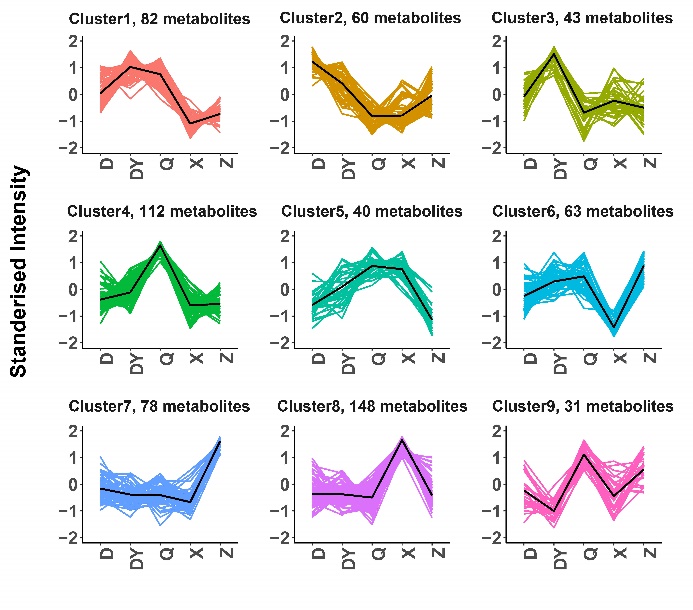


**Supplementary Figure 3** K-Means Plot of the differentially accumulated metabolites

A.


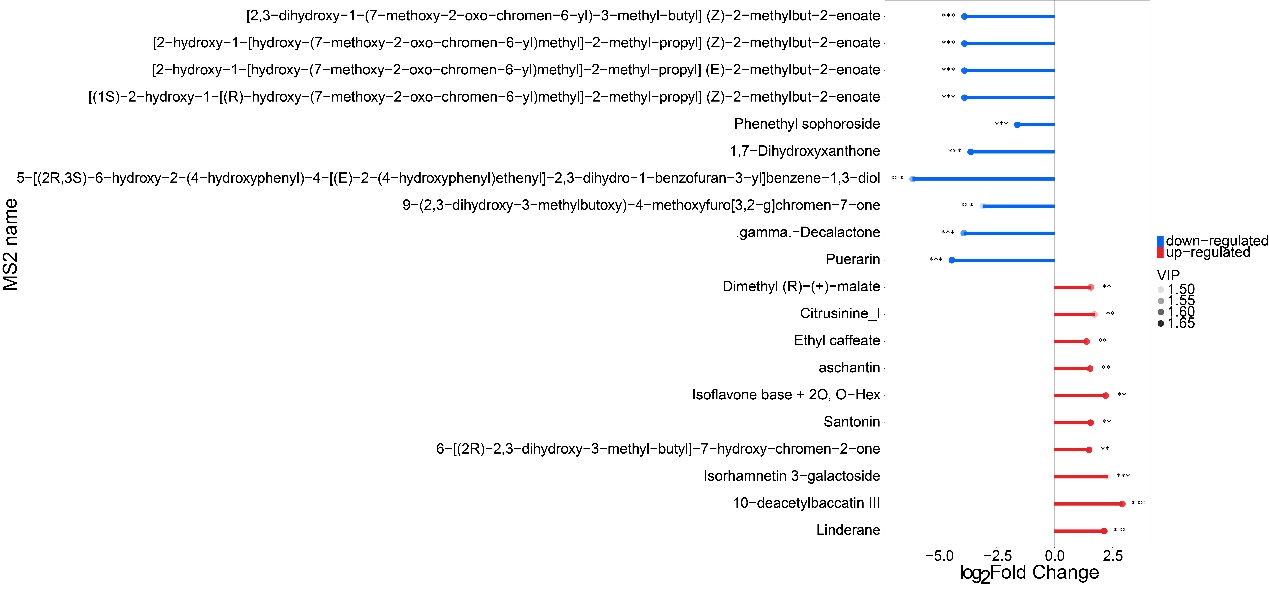


B.


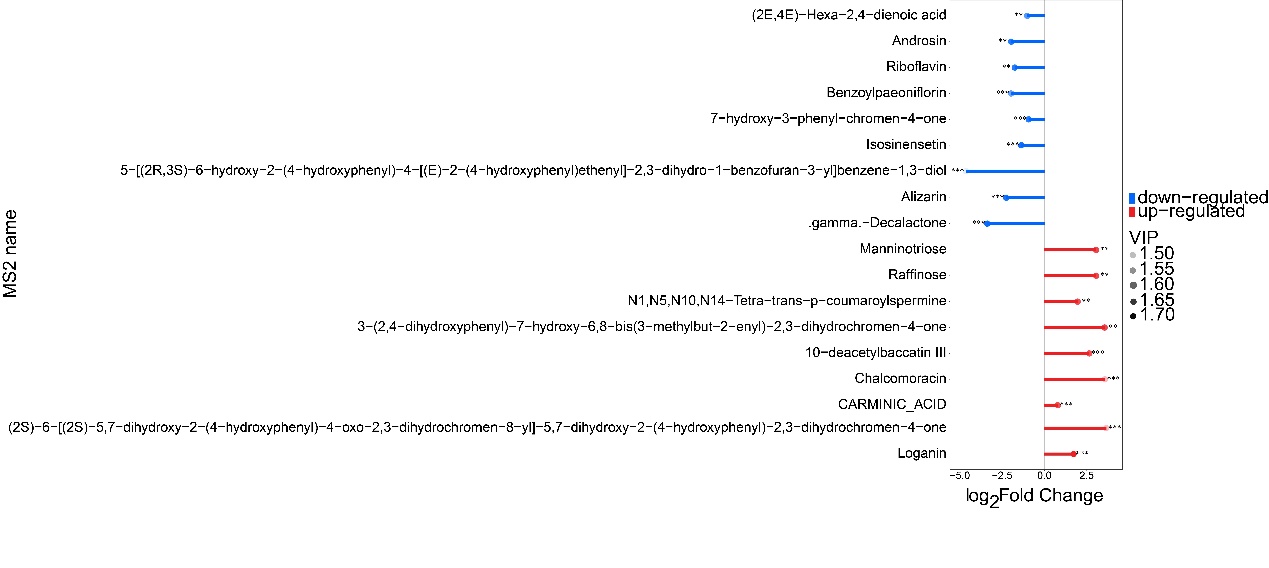


C.


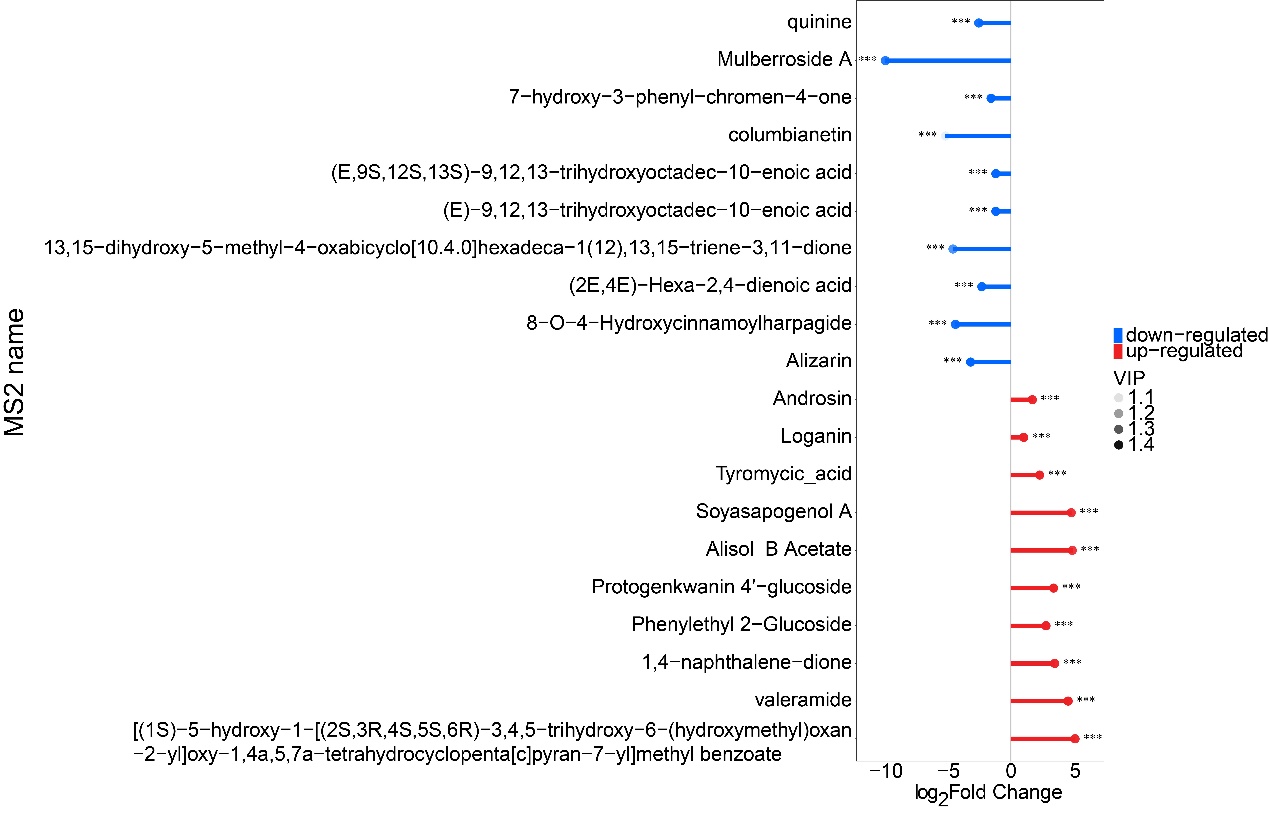


D.


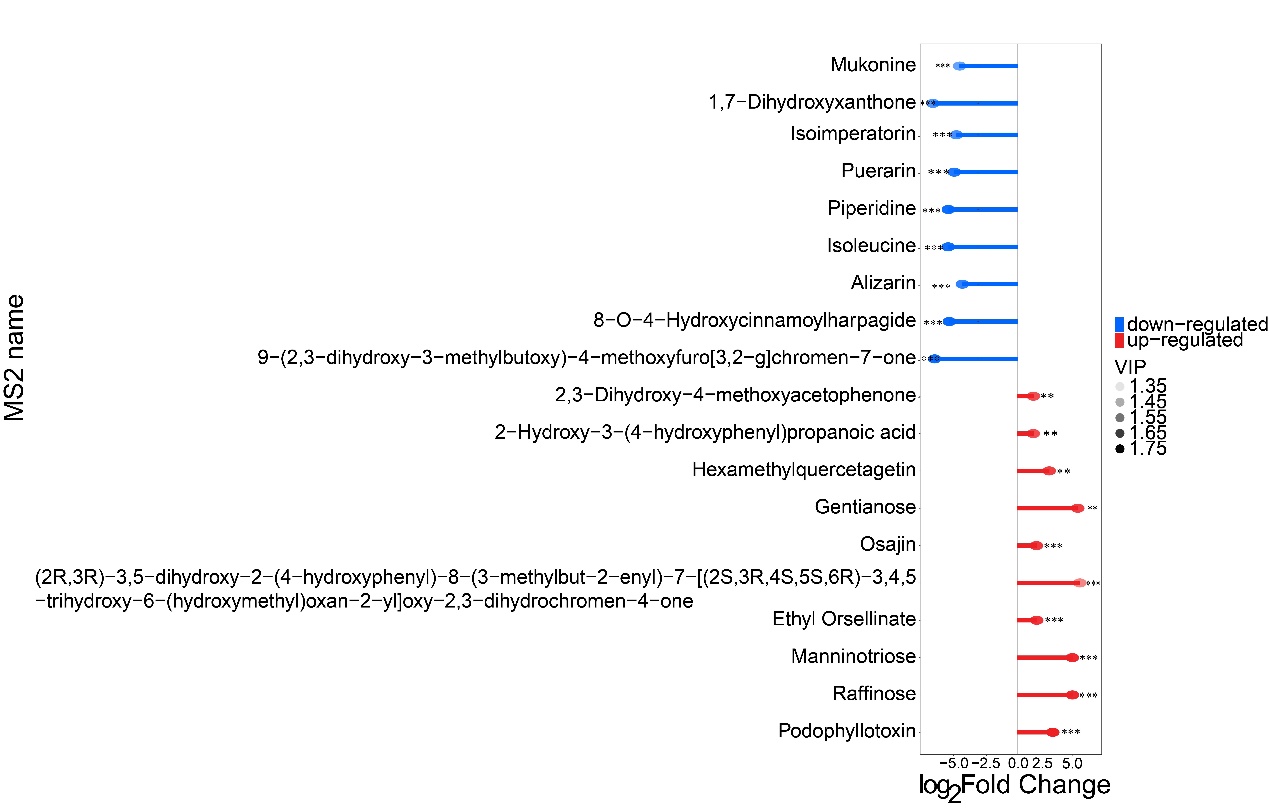


**Supplementary Figure 4** Matchstick map of significantly different metabolites (A. Q vs D; B. Q vs DY; C. Q vs X; D. Q vs Z)
